# Ayurveda, evidence-base and scientific rigor

**DOI:** 10.4103/0975-9476.72605

**Published:** 2010

**Authors:** Bhushan Patwardhan

**Affiliations:** *Editor-in-Chief, J-AIM*

Ayurveda describes five ways to acquire knowledge and create an evidence base. It’s concept of Apta means an unbiased and intuitive acquisition of knowledge from the masters; Pratyaksha refers to observational data acquired through the senses or their extensions in the form of actual experimentation; Anumana is evidence derived from logical analysis leading to a deduction; Upamana concerns evidence generated by analogy (or similar association between variables); while Yukti treats facts ascertained from a planned intervention to test an idea or evaluate an observation. Together, these reflect the epistemology of Ayurveda and its robust approach to ensure evidence based rigor.[1] Today, a primary need of Ayurveda is for transdisciplinary research designed to study its fundamental concepts, medicines and treatments. An earlier thought leadership article by J-AIM Editorial Board Chairman R. H. Singh rightly states, “Research is the prime need of contemporary Ayurveda, but modern research on Ayurveda has not been very rewarding for Ayurveda itself. Much of it uses Ayurveda to extend modern bioscience.”[2] It is important to develop intercultural standards for research in traditional medicine, with the right approach leading to appropriate methodology and study protocols.[3] The epistemological approaches to knowledge discovered by Ayurveda are systemic and holistic, whereas in the biomedical sciences they are more structural and reductionist. It is important to understand and respect both these realities. J-AIM is not a specialty journal concerned with a single discipline, but is devoted to quality transdisciplinary and translational research conducted without compromising the core concepts of any discipline, particularly applying this principle to Ayurveda. There is a growing realization that in the past, research on Ayurveda was often conducted wearing the spectacles of modern science, making assumptions that may not actually apply to Ayurveda itself. Design of preclinical or clinical research protocols should aim to conduct studies on Ayurveda, as it is actually practiced in a pragmatic or whole systems approach. Only then can the system be genuinely evaluated. Anything else is a compromise.

In this light, correspondence published in this issue about the transdisciplinary article by Priyadarshini *et al.*[4] has a special significance. We would like to thank M. S. Valiathan, an esteemed member of our Editorial Advisory Board, for sharing serious concerns expressed by S. C. Lakhotia about this paper (see pages 171–172, this issue). The authority of Lakhotia in the field of *Drosophila* research in India, and Valiathan’s significant contributions in medicine and appreciation toward Ayurveda are well known. After receiving communications from these esteemed scientists, as Editor-in-Chief, I took immediate steps to consult the Executive Editor, authors and reviewers to obtain more information. We studied the history of the article and obtained responses from reviewers and authors. I also discussed the article in detail with the Editor, who was responsible for processing this particular manuscript. The editorial policies and circumstances, which led to its final acceptance, are given here. We attempt fairly rigorous due diligence at J-AIM for all papers presented for review. We take into account a background and importance of the study and unique contributions made by authors. J-AIM has a sufficiently large number of competent reviewers from diverse disciplines and in this case they contributed significantly to improvements in the article. The article had undergone nearly 5 months peer review process after its submission on October 20, 2009, until its final acceptance on April 5, 2010.

The first author, Vaidya Priyadarshini, has been teaching at Government Ayurveda College, Mysore, for many years. She initiated this work in 2007 in collaboration with Asha Devi, who worked at the Drosophila Stock Center of University of Mysore for 17 years. Vaidya Priyadarshini and her collaborators claim to have tested several rasayanas specially formulated for *Drosophila*. They claim that their data on specially formulated rasayanas have produced comparable results on all *Drosophila melanogaster* lines tested. Priyasarshini and her co-authors responded responsibly to the peer review process, agreeing to position their work as a “Preliminary Study”. Priyadarshini requested the Executive Editor to protect details of the rasayana formulation because she wanted to protect the intellectual property. According to the authors, formulating a rasayana acceptable to *D. melanogaster* was an achievement in itself.

J-AIM is primarily a journal dedicated to transdisciplinary research on Ayurveda. The readership of J-AIM requires some background information, especially for biology related studies. This may justify the relatively long introduction, model description and information related to aging and Ayurveda. I personally agree that the article has many verbose expressions and the style is a little aggressive. But as an editorial policy, we do not impose a particular style on authors and we do give them reasonable freedom to express their point, provided there is sufficient evidence base and scientific support. As an editorial policy, we encourage authors to improve their manuscripts rather than rejecting them simply based on style or other such matters. This is strictly with the objective of providing opportunities for Ayurveda researchers to improve their skills, without compromising either the rigor of the review process or quality outcomes. We think that such hand-holding and encouragement is necessary in the Ayurveda and integrative medicine sector.

I must accept an editorial error of not mentioning this article as a “black box” experiment in accordance with “General Guidelines for Methodologies on Research and Evaluation of Traditional Medicine” (http://apps.who.int/medicinedocs/en/d/Jwhozip42e/6.3.html). This has been addressed by publishing an *Erratum* in this issue. We are publishing a letter from Lakhotia along with a response from Priyadarshini *et al*. (see pages 172–173, this issue), along with a relevant letter from Madan Thangavelu (see pages 173–174, this issue). These communications are self-explanatory. This debate suggests a need for basic orientation to Ayurveda when researching its concepts, therapies or medicines. The same is applicable when researchers from Ayurveda use modern biology protocols. History shows that research that does not consider basic epistemological differences has rarely benefitted science or shastra.

The experience of the last 9 months has led J-AIM to adopt a conscious policy of mentoring authors wherever necessary. We will soon announce a policy of special guidance for authors, under which individual assistance will be provided, e.g., for expert advice on protocols, professional help for correct identification and standardization of botanical materials, as well as for improving language and editing. J-AIM will also announce a new policy for transdisciplinary articles on Ayurveda, giving preference to those where at least one of the authors is an Ayurveda expert.

We are still learning our trade, and our website is now beginning to function as it should. J-AIM’s editorial policy is not to reject submissions outright for the need of improved English, style or scientific refinement. Rather, editors and reviewers will offer advice on all such aspects of a paper until promising submissions are brought up to the requisite standard. However, we do encourage discussions, debates and healthy criticism between experts from Ayurveda and other transdisciplinary areas on relevance and scientific value. Longer times between submission and publication may be a necessary price to pay to ensure the scientific rigor without compromising the epistemological reflections. We thank our authors and reviewers for being tolerant and also for their enthusiastic cooperation.
